# Tandem CAR-T cells targeting CD70 and B7-H3 exhibit potent preclinical activity against multiple solid tumors

**DOI:** 10.7150/thno.43991

**Published:** 2020-06-18

**Authors:** Meijia Yang, Xin Tang, Zongliang Zhang, Lei Gu, Heng Wei, Shasha Zhao, Kunhong Zhong, Min Mu, Cheng Huang, Caiying Jiang, Jianguo Xu, Gang Guo, Liangxue Zhou, Aiping Tong

**Affiliations:** 1 State Key Laboratory of Biotherapy, West China Hospital, West China Medical School, Sichuan University, Chengdu, Sichuan province, China.; 2 National Engineering Laboratory for Internet Medical Systems and Applications, The First Affiliated Hospital of Zhengzhou University, Zhengzhou, Henan province, China.; 3 Department of Neurosurgery, West China Hospital, West China Medical School, Sichuan University, Chengdu, Sichuan province, China.

**Keywords:** CD70, B7-H3, Chimeric antigen receptor T cell, Immunotherapy, Solid tumor

## Abstract

**Purpose:** Given that heterogeneous expression and variants of antigens on solid tumors are responsible for relapse after chimeric antigen receptor (CAR)-T cell therapy, we hypothesized that combinatorial targeting two tumor-associated antigens would lessen this problem and enhance the antitumor activity of T cells.

**Methods:** The co-expression level of CD70 and B7-H3 was analyzed in multiple tumor tissue samples. Further, two putative antigens were identified in The Cancer Genome Atlas and Gene Expression Profiling Interactive Analysis database. Two CD70 targeted CARs with different antigen binding domain, truncated CD27 and CD70 specific single-chain antibody fragment (scFv), were designed to screen a more suitable target-antigen binding moiety. Accordingly, we designed a bivalent tandem CAR (TanCAR) and further assessed the anti-tumor efficacy of TanCAR-T cells *in vitro* and *in vivo*.

**Results:** Our results indicated that co-expression of CD70 and B7-H3 was observed on multiple tumor types including kidney, breast, esophageal, liver, colon cancer, glioma as well as melanoma. The CD70 targeted CAR-T cells with binding moiety of CD70 specific scFv exhibit a higher affinity and antitumor effect against CD70^+^ tumor cells. TanCAR-T cells induced enhanced ability of cytolysis and cytokine release over unispecific CAR-T cells when encountering tumor cells expressing two target-antigens. Further, low doses of TanCAR-T cells could also effectively control the lung cancer and melanoma xenografts and improved overall survival of the treated animals.

**Conclusion:** TanCAR-T cells targeting CD70 and B7-H3 exhibit enhanced antitumor functionality and improve the problem of antigenic heterogeneity and variant in the treatment against solid tumor and melanoma.

## Introduction

Genetic modification T cells with chimeric antigen receptor (CAR) are emerged as a promising immunotherapeutic approach, which could trigger directly and indirectly anti-tumor response in absence of antigen presentation via MHC molecule. Although adoptive transfer of CAR-T cells has achieved great success in hematological malignancies treatments 1-3, the clinical effect of CAR-T cells against solid tumors has been much less rewarding. The mechanism explaining the discrepancy is multifactorial. One of the most important mechanisms was target antigen heterogeneity. Downregulation or mutation of target antigens and antigen deletion were common after the treatment of solid tumor cells 4. This phenomenon was detected in several clinical studies of unispecific CAR-T cell against solid tumor 5, 6. To address this problem, we designed a bivalent tandem CAR (TanCAR) targeted two pan-tumor-associated antigens, CD70 and B7-H3 (CD276), which could also apply for the immunotherapy of multiple types of solid tumor and melanoma.

CD70, ligand of CD27, was firstly detected on the surface of Hodgkin and Sternberg-Reed cells, and was identified as a surface-expressed member of tumor necrosis factor receptor superfamily 7, 8. Expression of CD70 is restricted to a small subset of lymphoid lineage including highly activated B and T cells, mature dendritic cells and natural killer cells 9. The aberrant expression of CD70 has been detected on hematological malignancies and also on some solid tumors, such as osteosarcoma, renal cell carcinoma, thymic carcinoma, nasopharyngeal carcinoma and glioblastoma 10-14. Several reports have also provided the evidence suggesting the therapeutic potential of CD70 targeted CAR-T cell 15, 16.

B7-H3, a type I transmembrane protein, is a member of the B7 superfamily molecules 17. The expression of B7-H3 maintains in a low level in normal tissues whereas it is aberrantly overexpressed in a wide variety of cancers including gastric cancer, pancreatic cancer, neuroblastoma, endometrial cancer, glioma, melanoma, lung cancer, ovarian carcinomas and prostate cancer, suggesting B7-H3 a promising immunotherapeutic target 18-21. And our previous study also reported the anti-tumor ability of B7-H3 targeted CAR-T cell against glioblastoma 22.

In this study, we report a bispecific CAR molecule incorporating antigen recognition domains for CD70 and B7-H3, joined in tandem. Our study provided the evidence of enhanced anti-tumor efficacy of TanCAR-T cell against multiple cancers expressing CD70 and B7-H3 *in vitro* and vivo. Such finding suggests CD70 and B7-H3 targeted TanCAR-T therapy might be a potential immunotherapeutic strategy.

## Material and Methods

### Tumor cell lines

The NCI-H460, A375, MDA-MB-435, 786-O, Fadu and K562 tumor cell lines were purchased from the American Type Culture Collection (ATCC). A375 cell line with B7-H3 gene knocked out was produced using a CRISPR-Cas9 system. We designed a gRNA to target the exon of the B7-H3 gene using online server and subcloned it into lentiCRISPR V2 vector (Addgene plasmid #52961). Target of B7-H3-gRNA: 5'-ATGCGTTGCCCTGTGCCAGC-3'. Cells were transduced with the lentivirus and screened by puromycin. One week after transduction, the cells were stained with B7-H3-specific monoclonal antibody (mAb)-J42, which was generated using the traditional hybridoma technique and B7-H3^-^ cells were then sorted using a fluorescence-activated cell sorter (FACS, BD Biosciences). For longitudinally monitoring tumor burden *in vivo*, NCI-H460 and A375 cells expressing firefly luciferase (NCI-H460.ffLuc and A375.ffLuc) were obtained by lentivirus transduction followed by puromycin selection using a lentivirus vector. The NCI-H460, A375, MDA-MB-435, A375^B7-H3 Ko^, NCI-H460.ffLuc and A375.ffLuc cell lines were maintained in DMEM, and 786-O and K562 cell lines were grown in RPMI-1640. All cell culture mediums were supplemented with 10% FBS (HyClone), 2mM L-glutamine, and 1% Penicillin-Streptomycin mixture (HyClone).

### Immunohistochemistry (IHC)

Commercial tissue microarray (Catalog no. HSki-C072PT-01, OD-CT-RsLug02-004, HLugA030PG02, OD-CT-UrKid02-001 and OD-CT-UrKid02-003) were purchased from the Shanghai Outdo Biotech company. IHC staining was performed according to an established protocol of Shanghai Outdo Biotech. Briefly, all the tissue microarrays were deparaffinized and blocked with 3% H_2_O_2_ in distilled water. Antigen retrieval was completed by immersing of slides in EDTA retrieval solution under pressure for 15 minutes. Blocking 5% goat serum was done in humidified conditions for 30 minutes at room temperature, followed by overnight incubation at 4°C with primary antibodies. Next day, slides were developed using a two steps detection kit and DAB chromogen (ZSGB-Bio), counterstained with hematoxylin (Biosharp). For the primary antibody, the murine anti-CD70 antibody (San Cruz Biotechnology Catalog no. sc-365539) and the rabbit anti-B7-H3 antibody (Cell Signaling Technology Catalog no.14058T) was used.

### Expression and purification of recombinant proteins

DNA sequences encoding human truncated CD27 (extracellular domain of CD27), CD70 extracellular domain, B7-H3 extracellular domain, CD70 specific scFv (derived from the CD70-16D_cc-scFv sequence, Patent number: WO2017021354A1) and B7-H3 specific scFv (derived from mAb-J42) were synthesized by GENEWIZ. And all the cDNA was sub-cloned into a pVAX1 based expression vector with human or murine IgG1 Fc and (His)_6_ tag fusion at the C-terminus, respectively. Transient expression in the HEK293T cell line was performed by using expression vectors and optimal DNA to PEI ratio was determined with 1:3. The cells were cultured in FreeStyle™ 293 Expression Medium (Thermo Fisher Scientific) for 4~5 days. Then the culture supernatants were harvested and centrifuged for 30 min at 10 000 × g, 4 °C. The recombinant proteins were initially purified by Ni-NTA column chromatography, and eluted with elution buffer (25mM Tris, pH 8.0, 250mM NaCl, 250mM Imidazole, 5% (v/v) glycerol and 1mM PMSF). The eluted recombinant proteins were then loaded to a Superdex200 gel filtration column (GE Healthcare) with gel filtration buffer (25mM Tris, pH 8.0, 250 mM NaCl, and 5% (v/v) glycerol, 1mM PMSF) followed by analysis of recombinant proteins purity through SDS-PAGE. Finally, recombinant proteins were concentrated and stored at -80 °C for later studies.

### Immunofluorescence

Tumor cell surface expression of CD70 and B7-H3 were detected using CD70 scFv.mFc and B7-H3 scFv.hFc chimeric proteins followed by Cy3-conjuncted goat anti-mouse Fc (Proteintech) for CD70 scFv.hFc and Alexa Fluor 594-conjugated goat anti-human Fc (Jackson ImmunoResearch) for B7-H3 scFv.hFc. Cells were blocked with 5% bovine serum albumin (BSA) and incubated with chimeric proteins for 2 hours at 4 °C. For secondary staining, cells were washed three times prior to 60 minutes incubation at 4 °C with secondary antibody in the dark. Microscopy images were captured using confocal microscope.

### Flow cytometry

Based on tdTomato expression levels, we acquired CAR-T cell transduction efficiency. CD8 expression on T cells was determined using CD8-APC (BD Biosciences). For tumor cell lines, CD70 expression was assessed using antiCD70 antibody (Santa San Cruz Biotechnology, G-7) followed by Alexa Fluor 594-conjuncted goat anti-human and B7-H3 expression was analyzed using a B7-H3 specific APC-conjugated antibody (BioLegend, MIH42). The cell samples were incubated with the responding antibody at 4 °C in the dark and then washed with PBS containing 2% FBS and 0.1% sodium azide. Before analysis, the samples were fixed in 0.5% paraformaldehyde. Flow cytometry analyses were performed using a FACSCalibur flow cytometer (BD Biosciences) and data were analyzed using FlowJo software.

### Construction of the TanCAR-encoding transgene

The CD70 specific scFv are previously described. The anti-B7-H3 scFv sequence were derived from a highly specific mAb against B7-H3 (clone: mAb-J42) generated by our group using standard hybridoma technique. The TanCAR-encoding DNA sequence was optimized using the GeneOptimizer software, aiming at obtaining maximum protein production. The TanCAR molecule consist of a CD8 leader, followed by CD70 specific scFv that is separated from B7-H3 specific scFv by a 15-amino acid glycine/ serine repeat linker, hinge domain, CD8 transmembrane, the signaling domain of the costimulatory molecule 4-1BB, the signaling domain of the T cell receptor CD3-zeta chain. A P2A ribosome skip sequenece separates the CAR sequence from a tdTomato as a CAR-T cell tracker. The encoding transgene was synthesized by GENEWIZ Gene Synthesis service and was sub-cloned into the lentiCRISPR V2 based lentiviral backbone without the puromycin resistance gene. Other CAR lentiviral expression vectors were also constructed in the same way.

### Lentivirus production and transduction of T cells

To produce lentiviral supernatant, HEK293FT cells were co-transfected with the CAR-encoding lentiviral plasmid, packaging plasmid psPAX2 (Addgene plasmid#12260) and pMD2.G (Addgene plasmid#12259), using transfection reagent PEI (Roche Applied Science). After 36h and 60h, the supernatants were harvested and removed any cell debris by filtering through a 0.45 µm filter, then centrifuged for 2h at 15000 rpm, 4 °C in order to acquire lentiviral pellet. The pellet was then resuspended in pre-cooling RPMI-1640 medium. Aliquots of lentivirus were stored at -80 °C and lentiviral particle titers were measured by transduction of HEK293T cells.

Peripheral blood mononuclear cells (PBMCs) from healthy and consenting volunteers were isolated by gradient centrifugation at 800g for 15min using Lymphoprep (Greiner Bio-One) at room temperature and cultured with T-cell media supplemented with 200ng/ml OKT3(Biolegend), 100ng/ml anti-CD28 mAb (Biolegend) and 100U/ml IL-2 (Life Science) at densities of 1×10^6^ cells/ml for 48 hours. Supernatants containing lentivirus were mixed with the activated human T cells (2×10^6^ cells/ml) in the presence of 1μg/ml polybrene(Sigma-Aldrich) followed by incubation for 12 hours in the presence of 100U/ml IL-2. The transduced T cells were collected and continuously cultured in medium containing IL-2. Transduction efficiency was determined by analyzing tdTomato expression on CAR-T cells.

### Cytotoxicity assays

The cytotoxic activities of CAR-T cells were assessed by a standard ^51^chromium (Cr)-release assay. Briefly, tumor cells were labeled with 100μCi of ^51^Cr for 1h at 37 °C, washed three times and used as target cells. Cells at various effector-to-target (E:T) cell ratios were added in triplicate to wells of a 96-well conical plate together with 5×10^3^ target cells. Cytotoxicity assays were performed for 4h at 37 °C. Following incubation, cell-free supernatants were harvested and released ^51^Cr was measured in a gamma counter. The mean percentage of specific lysis of triplicate wells was determined using the following formula: (test release - spontaneous release) / (maximal release - spontaneous release) × 100.

### Analysis of cytokine production

CAR-T cells were cocultured with tumor targets at an effector-to-target ratio of 2:1 in a 24-well plate. Following 24 hours of coculture at 37 °C, coculture supernatants were collected and IFNγ and IL-2 release was measured by ELISA in accordance with the manufacturer's instructions (Thermo Fisher Scientific).

### Animal studies

All animal experiments were performed following the protocol approved by the Biomedical Ethics Committee of the West China Hospital, Sichuan University. Recipient NSG mice, aged 6-7 weeks, were female and purchased from GemPharmatech Company. For A375 tumor models, mice were injected subcutaneously in the right hind flank with 1×10^6^ A375 cells expressing firefly luciferase (A375.ffLuc) cells in PBS on day 0. For NCI-H460 tumor models, mice were injected with 5×10^5^ NCI-H460 expressing firefly luciferase (NCI-H460.ffLuc) cells. For CD70^+^/B7-H3^-^, CD70^-^/B7-H3^+^ and CD70^-^/B7-H3^-^ control tumor models, mice were injected subcutaneously with 3×10^6^ Fadu cells expressing firefly luciferase (Fadu.ffLuc), 1×10^6^ A375^B7-H3 Ko^ cells expressing firefly luciferase (A375^B7-H3 Ko^.ffLuc), and 1×10^6^ K562 cells expressing firefly luciferase (K562.ffLuc), respectively. The progressively growing xenografts of mice were evidenced by bioluminescence signal. Mice with growing xenografts were randomized to treatment groups and received tail-vein injection of different doses of CAR-T cells on day 5-7 following tumor inoculation. Tumor volume was calculated as follows: tumor size = long diameter (short diameter2)/2.

### *In vivo* bioluminescence imaging

To monitor tumor growth, mice were anesthetized by isoflurane and injected intraperitoneally D-luciferin potassium salt (Beyotime) suspended in PBS with 150mg/kg. The mice were imaged using an IVIS Spectrum Imaging System (Caliper Life Sciences) 15 minutes after injection. The bioluminescence image was acquired and quantified in the region of interest by Living Image software (Caliper Life Sciences).

### Statistical analysis

The date, including cytotoxicity assays, ELISA, and survival analysis, were summarized using descriptive statistics and statistical analysis was performed with GraphPad Prism software7.0. Data are presented as means ± standard deviation (SD). Statistically significant differences were evaluated by Student's t-test comparing two experimental groups. The survival curves evaluating the tumor-bearing mice were constructed using the Kaplan-Meier method and statistical differences were determined by the log-rank testing. * p<0.05 was considered to indicate a significant difference.

### Study approval

All animal experiments followed a Protocol (2017-151) approved by the Biomedical Ethics Committee of the West China Hospital of Sichuan University. Blood samples from healthy donors and commercial tissue microarray from tumor patients were also approved by the Biomedical Ethics Committee of the West China Hospital of Sichuan University (Ethical Approval Document: 2018-061). Written informed consent was obtained from donors and patients.

## Results

### CD70 and B7-H3 expression profiles of tumor tissues and tumor cell lines

**Figure 1:**
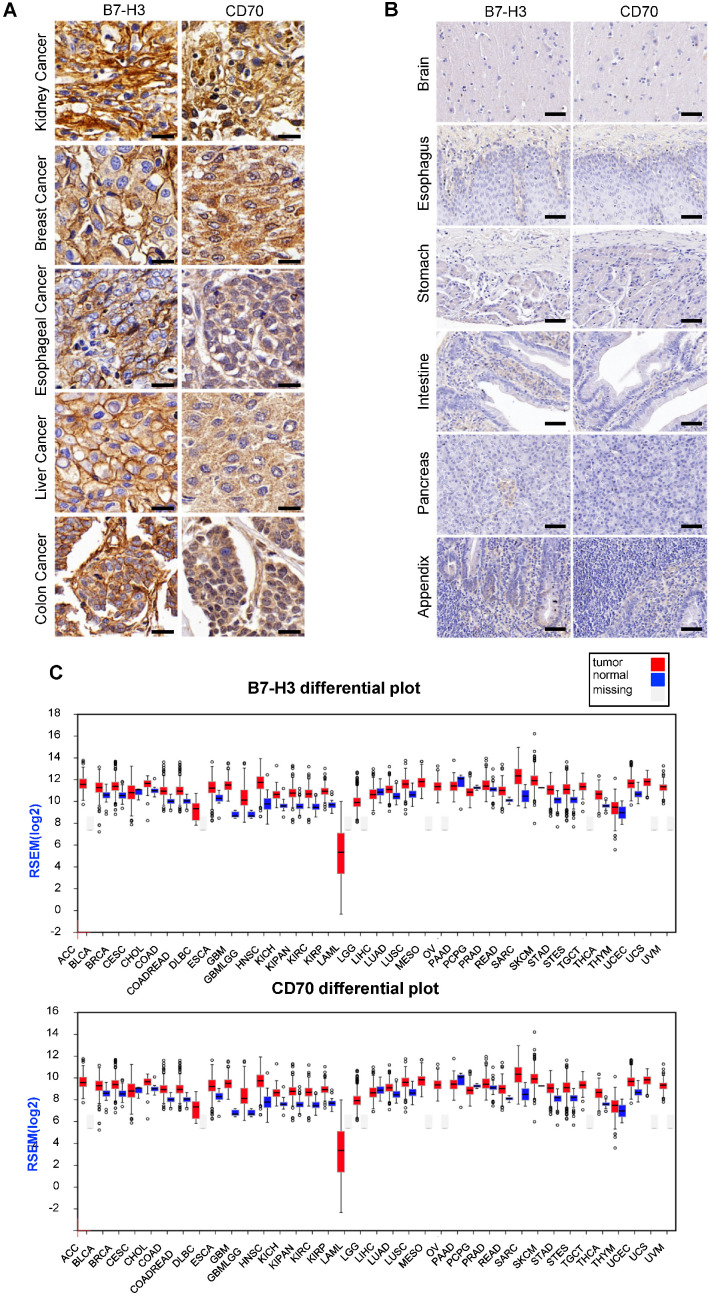
Expression of CD70 and B7-H3 on human tumor tissues. (A) Representative images of IHC staining of CD70 and B7-H3 on human tumor tissue microarrays were shown. (Scale bar, 20 μm) (B) IHC result of CD70 and B7-H3 staining in normal tissues including brain, esophagus, stomach, intestine, pancreas, appendix. The representative images were shown. (Scale bar, 50 μm) (C) Differential expression profile analysis of B7-H3 and CD70 in tumor and normal tissues based on the TCGA database.

For detecting the expression of B7-H3 and CD70, multiple tumor microarrays including kidney, breast, esophageal, liver and colon cancer as well as melanoma, glioma and normal tissues specimen were stained by the method of IHC. The results indicated that most of tumor tissues were B7-H3 or CD70 positive. A portion of tumor cases were highly co-expressed B7-H3 and CD70, including 2 of 5 melanoma cases, 19 of 62 lung cancer cases, 16 of 64 kidney cancer cases, 5 of 15 liver cancers cases, 22 of 62 breast cancer cases, 13 of 32 esophageal cancer cases, 17 of 32 colon cancer cases and 7 of 33 glioma cases (Table S1), while there was no detectable B7-H3 or CD70 expression in normal tissues. Representative case images of multiple tumor and normal tissues were shown in Figure 1A and Figure S1. Further, based on the RNA-seq analysis from the Cancer Genome Atlas (TCGA) and Oncomine database, we found transcripts of CD70 or B7-H3 were up-regulated in multiple cancer types which includes not only the tumor types mentioned above but also cervical, neck, pancreatic cancer as well as leukemia and lymphoma (Figure 1B, S2-3). Differential expression profile analysis based on Gene Expression Profiling Interactive Analysis (GEPIA) suggested that the expression of CD70 or B7-H3 were also up-regulated on multiple tumors, compared with the corresponding normal tissue (Figure 1C).

**Figure 2:**
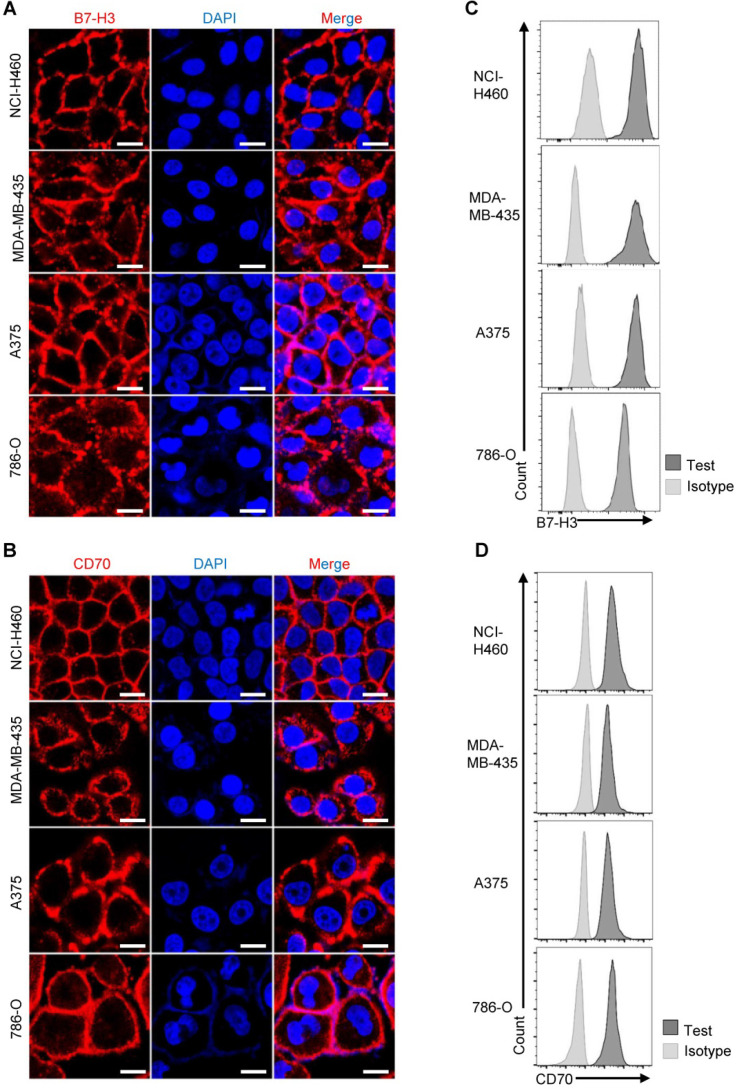
Expression of CD70 and B7-H3 in human solid tumor cell lines. (A, B) Representative images showed the immunofluorescence staining of B7-H3 and CD70 together with DAPI in NCI-H460, A375, MDA-MB-435 and 786-O tumor cells. (Scale bar: 20 μm) (C, D) Flow cytometry result indicated high expression of CD70 and B7-H3 on the four solid tumor cell lines.

Likewise, we detected the expression of CD70 and B7-H3 among various tumor cell lines. Examined by immunofluorescence using purified human B7-H3 scFv-mFc and CD70 scFv-mFc fusion protein as primary antibody, expression of CD70 and B7-H3 could be detected on the surface of NCI-H460, A375, MDA-MB-435 and 786-O tumor cells (Figure 2A-B). Consistent with immunofluorescence result, flow cytometry results also suggested that CD70 and B7-H3 was highly expressed in these cell lines (Figure 2C-D). Further, we also determined the CD70^-^/B7-H3^+^ (Fadu) and CD70^-^/B7-H3^-^ (K562) cells. In follow-up analysis the specific anti-tumor effect of TanCAR-T cells, we served Fadu and K562 cells as CD70^-^/B7-H3^+^ and CD70^-^/B7-H3^-^ control, respectively. Since we could not identify a CD70^+^/B7-H3^-^ solid tumor cell line, we establish the CD70^+^/B7-H3^-^ control cell line by knocking out B7-H3 gene in A375 cells using CRISPR/Cas9 technique. The immunofluorescence result of the control cell lines and sequencing analysis of gene-editing were provided in Figure S4.

### Generation of CAR-T cells

**Figure 3:**
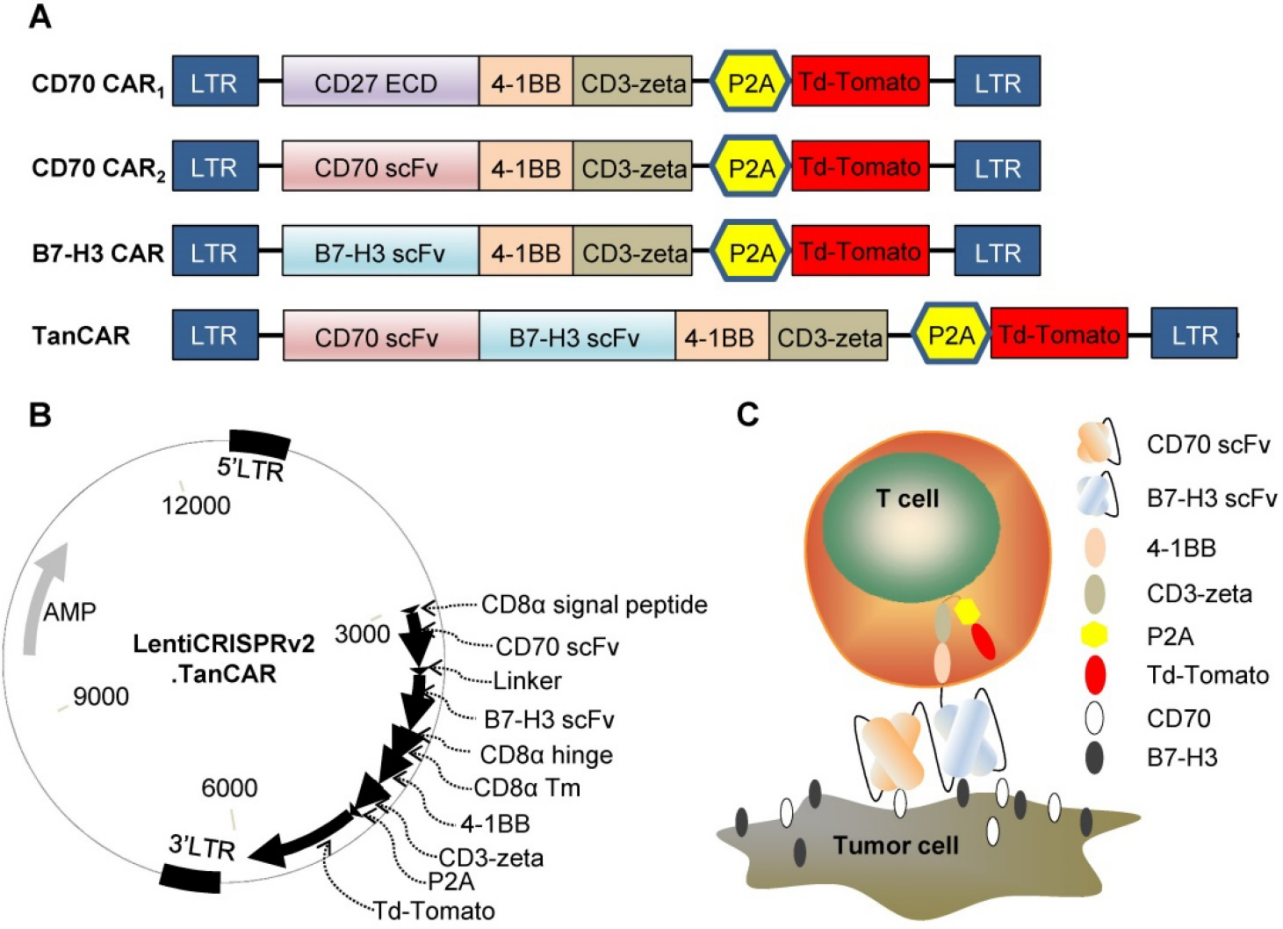
Construct of CAR (A) Schematic diagrams showing the composition of the four CARs used in this study: CD70 CAR_1_, CD70 CAR_2_, B7-H3 CAR and TanCAR. (B) The lentiviral backbone plasmid encodes the TanCAR. (C) Cartoon depicted of TanCAR targeting respective tumor antigens.

To redirect the specificity of T cells towards both CD70 and B7-H3 simultaneously using a single CAR molecule, we generated a TanCAR: a bivalent CAR molecule that can target 2 tumor-associated antigens in a tandem structure. The ectodomain of TanCAR consists of CD8α signal peptide, CD70-specific scFv, 15-amino acid glycine/ serine repeat linker, B7-H3-specific scFv and hinge domain. The transmembrane and intracellular domain includes CD8α transmembrane domain, cytoplasmic domain of 4-1BB/CD3ζ, P2A and tdTomato (Figure 3A). Further, we designed two different CD70 targeted CARs with different antigen binding domain, truncated CD27 (trCD27: the extracellular binding portion of CD27) and CD70 specific scFv, to investigate which of the component, trCD27 and CD70 specific scFv, was more suitable for being the antigen binding domain of CD70 targeted CAR. All encoding genes mentioned above were codon-optimized, synthesized and sub-cloned in a lentivirus vector (Figure 3B). A model illustrating docking of the TanCAR-T cell to B7-H3 and CD70 positive tumor cell was shown in Figure 3C.

**Figure 4:**
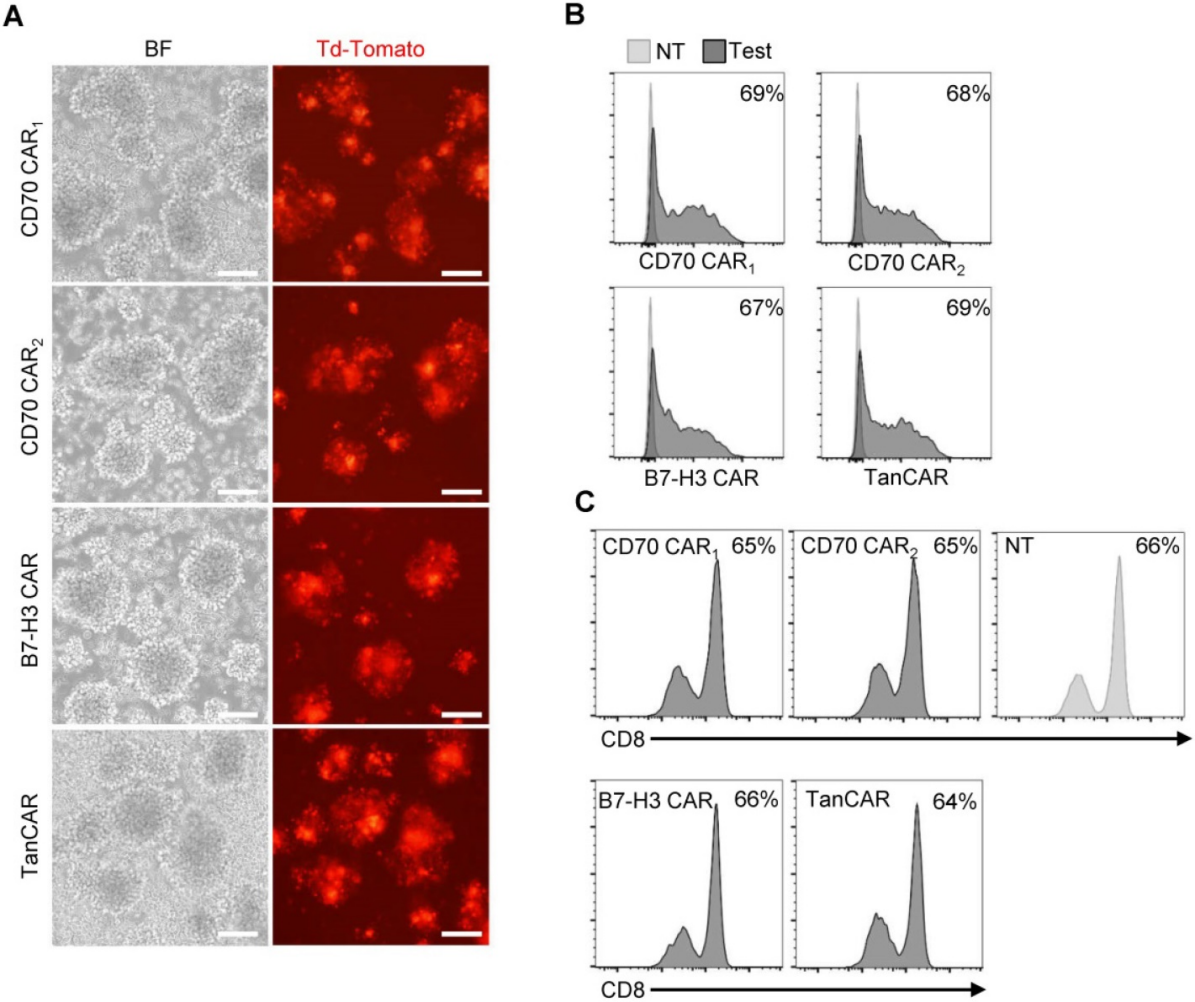
Generation of CAR-T cells. (A) Images of transduced CAR-T cells were captured using inverted fluorescent microscope. (Scale bar: 100 μm) (B) The transduction efficiency was measured by tdTomato positive cells using flow cytometric analysis. (C) Flow cytometry results illustrated the frequency of CD8^+^ T cells on 7 days post-transduction, compare with the non-transduced T cells.

The transduction efficiency was examined based on tdTomato co-expression levels (Figure 4A). By flow cytometry, we determined >60% of T cell transduced. In the subsequent assay, the TanCAR transduction was normalized to unispecific CAR-T cells (Figure 4B). Simultaneously, CD8 subset of T cells was measured and the ratios of CD8^+^ T cells were not significantly altered between the transduced and non-transduced T cells (Figure 4C).

### TanCAR-T cells distinctly recognize CD70 and B7-H3 target antigens and exhibit improved effector functions *in vitro*

**Figure 5:**
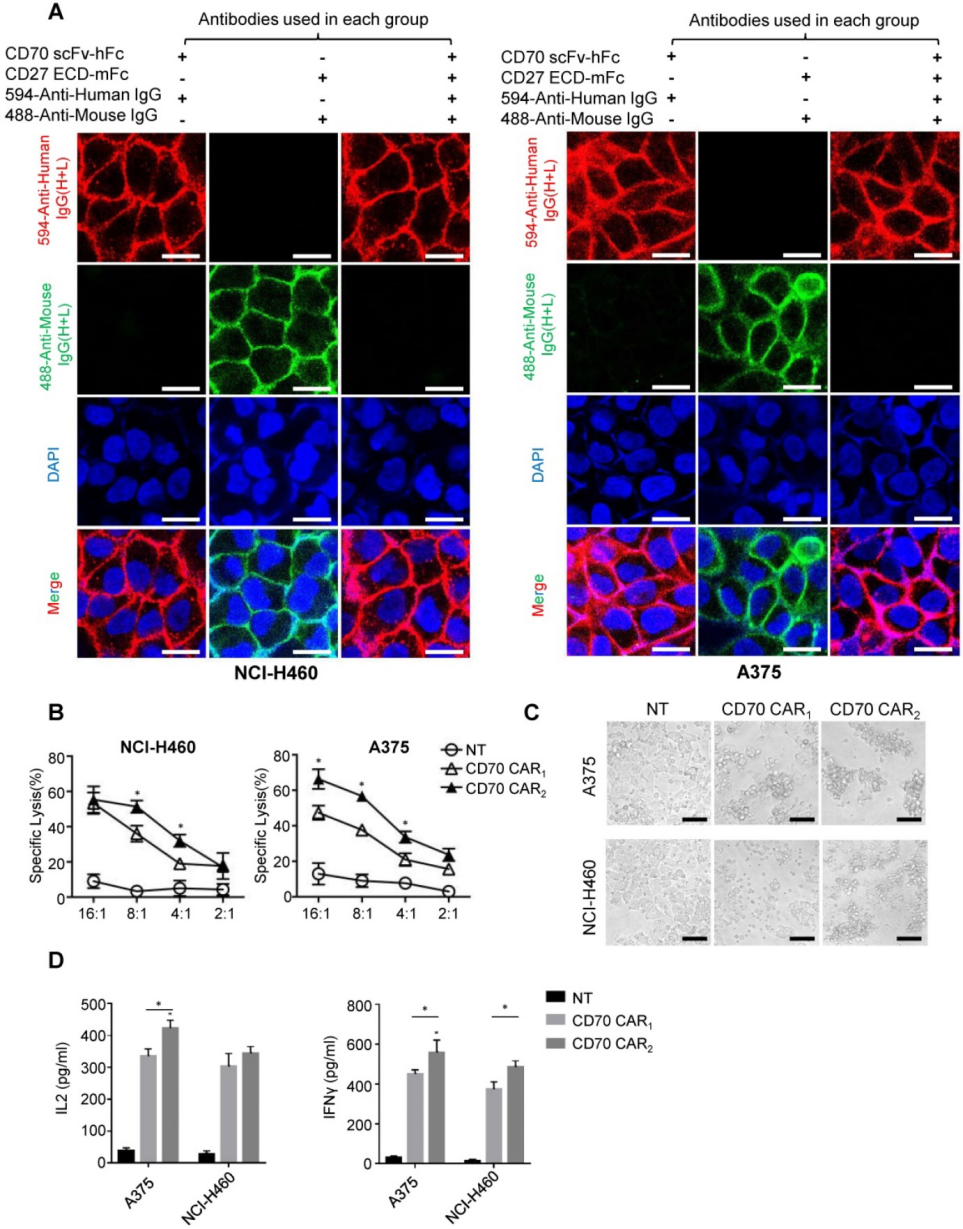
Functional analysis of CD70 CAR_1_ and CD70 CAR_2_. (A) To assess the affinity of two CD70 binding fragments, immunofluorescence was performed using human trCD27.mFc and CD70 scFv.hFc chimeric protein as the primary antibody. Images showed the immunofluorescence staining of CD70 by NCI-H460 and A375 tumor cells. (Scale bar: 20 μm) (B) 4-hour ^51^Cr cytotoxicity assays indicated a higher tumor killing of CD70 CAR_2_-T cells against target cells. (C) Microscopy images were captured 8 hours after A375 or H460 cells cocultured with CD70 CAR_1_, CD70 CAR_2_ and NT T cells at a ratio of 2 effector cell to 1 target cells. (Scale bar: 50 μm) (D) ELISA results showed the IFN-γ and IL-2 secretion levels by CD70 CAR_1_, CD70 CAR2 and NT T cells encountering A375 or H460 cells.

Before the functional test, we identified the antigen binding domain applied for ectodomain CD70 targeted CAR. Human tumor cell lines NCI-H460 or A375 uniformly expressing CD70 was used to detect the affinity of trCD27 and CD70 specific scFv by immunofluorescence. As shown, the affinity of CD70 scFv (used in CD70 CAR_2_) chimeric protein for CD70 was higher than that of trCD27 (used in CD70 CAR_1_) chimeric protein (Figure 5A). For *in vitro* functional analysis, CD70 specific scFv CAR (CD70 CAR_2_) T cells exhibited distinctly antitumor efficacy compared with the trCD27 CAR (CD70 CAR_1_) T cells (Figure 5B-C). Similarly, higher cytokine (IFN-γ and IL-2) secretion level was observed in CD70 CAR_2_-T cells co-culturing with A375 cells (Figure 5D). Antigen binding domain of CD70 CAR_2_ was thus used in the following study.

**Figure 6:**
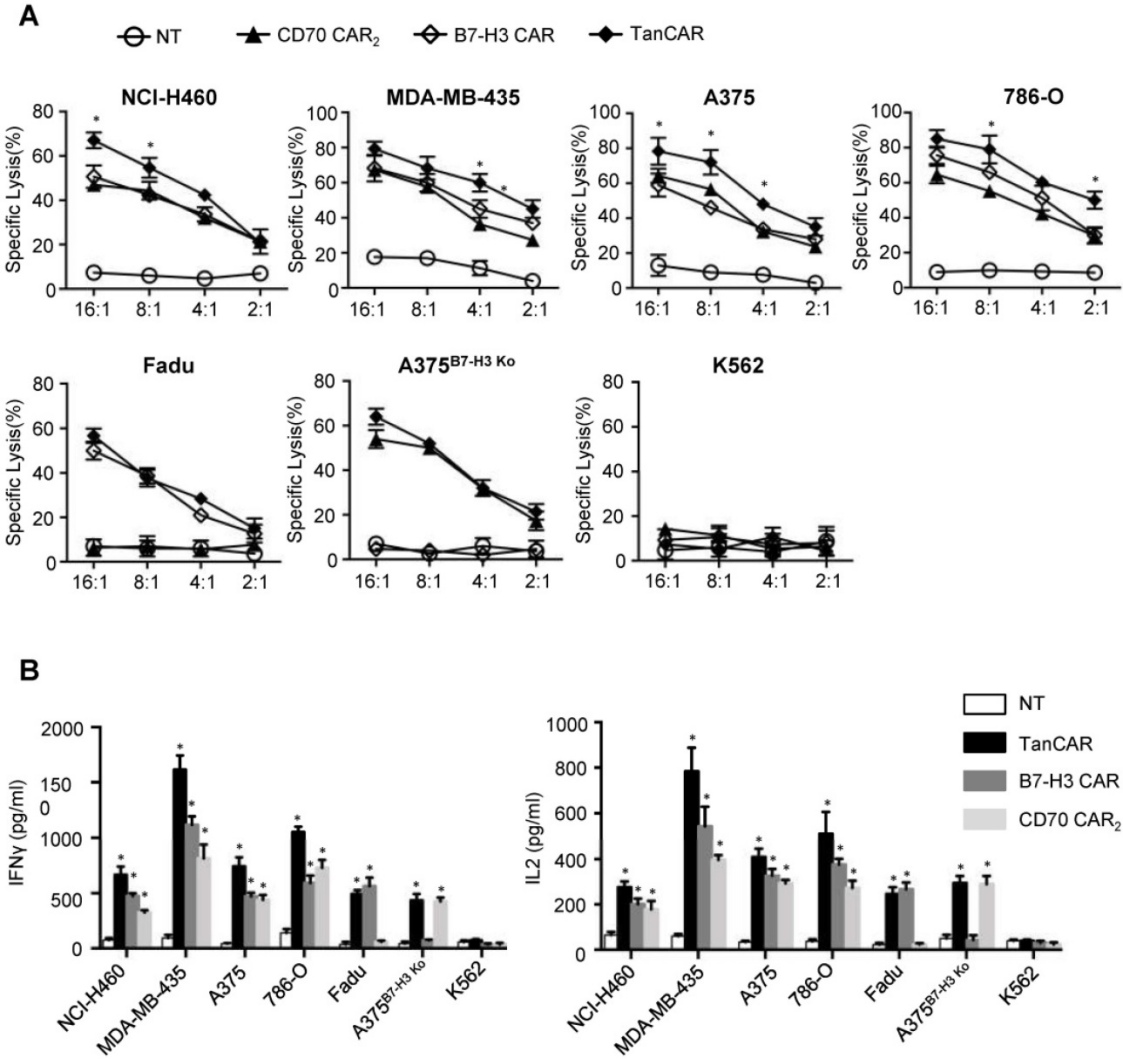
Activity of TanCAR-T cells against tumor cells expressing CD70 and/or B7-H3. (A) Four-hour ^51^Cr cytotoxicity assays of TanCAR-T cells against tumor cells expressing CD70 and/or B7-H3, compared with unispecific CAR and NT T cells. (B) Analysis of IFNγ and IL2 secretion level from supernatants of co-cultures of TanCAR, B7-H3 CAR, CD70 CAR_2_ and NT T cells with multiple tumor cells, as detected by ELISA. Shown are pooled data from 3 independent experiments done in triplicates.

For analysis of anti-tumor efficacy, NCI-H460, A375, MDA-MB-435 and 786-O were served as CD70^+^/B7-H3^+^ cell lines. Fadu, A375^B7-H3 Ko^, K562 were served as CD70^-^/B7-H3^+^, CD70^+^/B7-H3^-^ and CD70^-^/B7-H3^-^ control cell lines, respectively. As shown by ^51^Cr cytotoxicity assay, significantly higher killing effect at most effector to target ratios were observed in TanCAR-T cells against NCI-H460, A375, MDA-MB-435 and 786-O cells compared with unispecific CAR-T cells (Figure 6A). Further, TanCAR-T cells exhibited modest cytolytic activity on Fadu and A375^B7-H3 Ko^ cells, which was almost consistent with that of unispecific CAR-T cells, but not on K562 cells, indicating the specific anti-tumor response of TanCAR-T cells. For a more intuitive view of anti-tumor effect, we performed a coculture assay to compare TanCAR-T cells with control CAR-T cells when exposed to NCI-H460, A375, MDA-MB-435 and 786-O cell lines (Figure S5). In order to evaluate the relative cytokine serection capacity, we collected the supernatants for the detection of IFNγ and IL-2 secretion levels after 24 hours coculture of tumor and CAR-T cells. The secretion level was significantly higher in supernatants in samples with TanCAR T cells over unispecific CAR-T cells, while neither tumor cells cocultured with NT T cells nor K562 cocultured with TanCAR T cells had detectable levels of cytokines (Figure 6B).

### TanCAR-T cells exhibit enhanced antitumor activity *in vivo*

**Figure 7:**
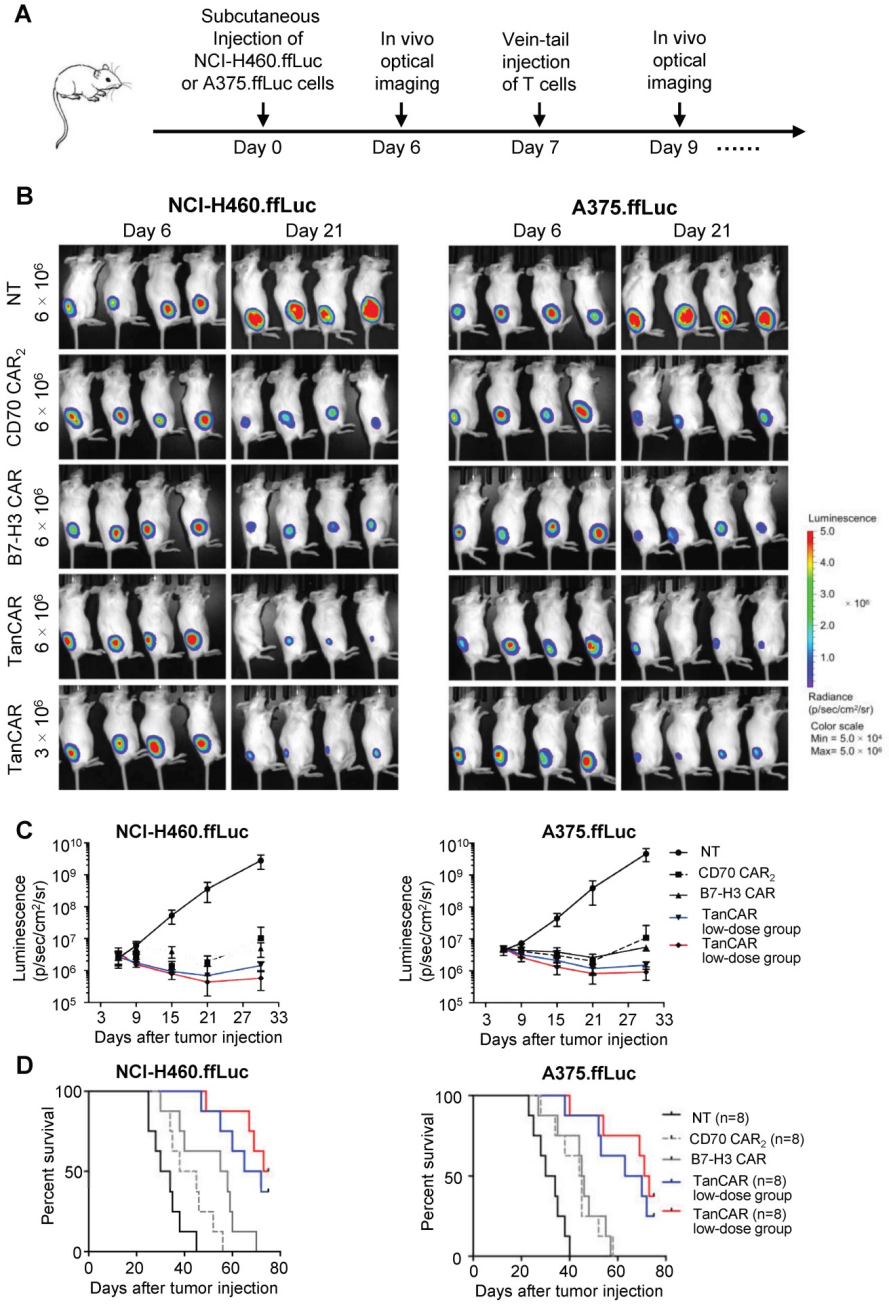
Antitumor response of TanCAR-T cells against CD70 and B7-H3 positive tumors *in vivo*. (A) The treatment scheme showed the timing of subcutaneous injection of tumor cells, vein-tail injection of CAR-T cells T cells and *in vivo* optical imaging. (B) Antitumor response of TanCAR-T cells in human subcutaneous xenograft models. NCI-H460.ffLuc or A375.ffLuc tumor bearing (confirmed by imaging 6 days after tumor implantation, 8/group) mice were adoptively transferred through tail vein injection with NT, CD70 CAR_2_, B7-H3 CAR or high/low doses (5×10^6^ or 1×10^6^/mouse) of TanCAR T cells on 7 days and 10 days post tumor inoculation. (C) Tumor growth was measured weekly by using Living Image software, and mean values per treated group were shown. (D) Kaplan-Meier survival curve were performed 75 days after T cells injection. Mice treated with TanCAR-T cells had a significantly longer survival probability in comparison with mice with NT, CD70 CAR_2_ or B7-H3 CAR-T cells.

Based on antitumor reactivity of these CARs *in vitro*, we thus assessed their antitumor ability against established tumor xenograft in two mouse model of human lung cancer and melanoma. In order to enable longitudinal monitoring of tumor burden, NCI-H460 and A375 cells expressing firefly luciferase (NCI-H460.ffLuc, A375.ffLuc) were obtained by transducing a luciferase-expressing construct. In this experiment, 5×10^5^ NCI-H460.ffLuc or 1×10^6^ A375.ffLuc cells were injected subcutaneously into NSG mice in the right hind flank. Tumors were allowed to establish for seven days and then NSG mice were intravenously injected with NT, CD70 CAR_2_, B7-H3 CAR or TanCAR-T cells on day 7 post tumor inoculation. General protocol schema is shown in Figure 7A. Tumor growth was monitored via the luciferase signal by *in vivo* optical imaging system (IVIS) over the course of 21 days. Tumor regression was observed in four groups treated by CAR-T cell as shown in Figure 7B-C. As expected, two models of human lung cancer and melanoma treated with TanCAR-T cells showed a more significant decrease in tumor burden, comparable to NT, CD70 CAR_2_ and B7-H3 CAR-T cells treated groups. The overall survival of the NCI-H460.ffLuc or A375.ffLuc tumor-bearing mice was significantly prolonged in the high and low dose of TanCAR-T cells treated group (Figure 7D). To further assess the specific antitumor efficacy of TanCAR-T cells, we performed another *in vivo* experiment wherein established xenografts of tumor expressing CD70^-^/B7-H3^+^, CD70^+^/B7-H3^-^ and CD70^-^/B7-H3^-^ (Fadu, A375^B7-H3 Ko^ and K562 cell lines) were treated with TanCAR and NT T cells. As shown, TanCAR-T cells induced tumor regression in Fadu and A375^B7-H3 Ko^ xenografts, but not in K562, proved by living imaging and tumor growth curve (Figure S6A-B). Collectively, these *in vivo* experiments indicated that TanCAR-T cells improve the control of established xenografts of tumor expressing CD70 and/or B7-H3 target antigens.

## Discussion

In this study, we construct a tandem CAR molecule targeting 2 tumor-associated antigens, B7-H3 and CD70, and found that TanCAR-T cells distinctly recognize the antigens and exhibited superior antitumor effect when encountering both antigens simultaneously. Further, TanCAR-T cells could also specifically target and kill tumor cell expressing single target antigen. In preclinical model of human lung cancer and melanoma, this bivalent targeting CAR-T cell could not only induce a more superior antitumor effect but also induce regression of tumor in a lower dose than unispecific CAR-T cells.

Adoptive transfer of CAR-T has exhibited extraordinary antitumor response in treating B cell cancer. This successfully application was achieved by targeting CD19, a B-cell lineage maker which is uniformly expressed in the cancer cells 23. However, because of the variable extents of antigenic heterogeneity in solid tumor tissues, it is difficult for the selection of single antigen as a universal target for CAR-T therapies 4, 24. One means to both broaden the target range of CAR-T cells and to target multiple malignancies with greater effect is to include two antigen binding domains in a single CAR structure. Thus, we chose two tumor-associated antigens, B7-H3 and CD70, as the targets for CAR-T therapy in our study. These two antigens were both over-expressed on multiple solid tumors, including brain, kidney, breast, liver, esophageal and colon cancer. Further, we and others have also reports the potential antitumor ability of CD70 or B7-H3 unispecific CAR-T cells in multiple preclinical models, which provided the foundation for the application of TanCAR-T therapy in the treatment of different solid tumor types 15, 16, 22, 25.

In our study, TanCAR-T cells distinctively recognized either B7-H3 or CD70 and enhanced their effector function as judged by tumor-lytic activity when both targets were encountered simultaneously while stimulation of unispecific CAR-T cells only resulted in suboptimal activity. Likewise, simultaneous encounter of both antigens mediated significantly higher cytokine secretion by TanCAR-T cells than did exposure to single target alone. Moreover, the TanCAR-T cells could induce a significant decrease in tumor burden in a relatively low dose, compared to the unispecific CAR-T cells, *in vivo* experiments. Although TanCAR-T cells could not eradicate the tumor completely, it did prolong the overall survival of tumor-bearing mice in comparison with control CAR-T cell treated group. Several reports also explain the superiority of TanCAR-T cells to unispecific CAR-T counterparts, including enhanced signaling and the ability to induce a robust immune synapse 26-30. This super-additive antitumor function pointed out a potential functional capacity of TanCAR-T cells upon the simultaneous antigenic stimulation. In addition, considering the low doses of effector cells, the infusions of TanCAR-T cells may be insufficient to eliminate the tumor completely. As future studies evaluating the TanCAR-T therapy, it will be worthwhile to assess the infusion doses that could induce tumor regression effectively. Also, further analysis of antigen loss in tumor-bearing mice treated with TanCAR-T cells may prove beneficial.

Previous studies suggested that affinity of the antigen recognition domain affects CAR T-cells efficacy. High and specific affinity of antigen recognition domain enhances antitumor function of CAR-T cells. Since several reports about CD70 targeted CAR-T cell therapy utilized CD27 as the antigen binding domain 15, 16, 31. In order to optimize the extracellular CD70 binding domain, we constructed two CD70-specific CARs using CD70 receptor (trCD27) or the scFv domain derived from a CD70-specific monoclonal antibody as the binding domain and assessed their affinity as well as antitumor efficacy *in vitro* and vivo. The result suggested the potential antitumor function of using the anti-CD70 scFv domain in the CD70 targeted CAR.

In summary, we have shown in a cohort of tumor types that overexpressed both CD70 and B7-H3, and we developed a TanCAR construct which might be applied in treating multiple solid tumors and melanoma. TanCAR-T cells exhibited enhanced antitumor activity and better tumor control in several preclinical models.

## Supplementary Material

Supplementary figures and tables.

## Abbreviations

CAR: chimeric antigen receptor
TanCAR: tandem chimeric antigen receptor
IHC: immunohistochemistry
mAb: monoclonal antibody
scFv: single-chain antibody fragment
hFc: human fragment crystallizable
mFc: mouse fragment crystallizable
PBMC: peripheral blood mononuclear cells
trCD27: the extracellular binding portion of CD27
NT: non-transduced
E:T: effector-to-target
ELISA: enzyme linked immunosorbent assay
TCGA: Cancer Genome Atlas
GEPIA: Gene Expression Profiling Interactive Analysis
