# Supplementary material for: Tandem CAR-T cells targeting CD70 and B7-H3 exhibit potent preclinical activity against multiple solid tumors
Source: Theranostics. 2020 Jun 18;10(17):7622–34. doi: 10.7150/thno.43991 (PMC7359081; doi:10.7150/thno.43991)
Supplement: Supplementary file 1 — Supplementary figures and tables. [file thnov10p7622s1.pdf]

Analysis Type by Cancer	Cancer vs. Normal B7-H3		Cancer vs. Normal CD70	
Bladder Cancer				
Brain and CNS Cancer	1			
Breast Cancer	3			3
Cervical Cancer			1	
Colorectal Cancer				
Esophageal Cancer	1			
Gastric Cancer	1			
Head and Neck Cancer			1	1
Kidney Cancer	1		2	
Leukemia			3	
Liver Cancer				
Lung Cancer				
Lymphoma			3	
Melanoma				
Myeloma				
Other Cancer				
Ovarian Cancer				
Pancreatic Cancer	1			
Prostate Cancer				
Sarcoma				
Significant Unique Analyses	8		10	4
Total Unique Analyses	314		436	

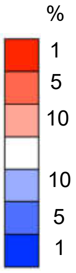

Figure S1. The graphic shows transcription level of B7-H3 and CD70, collected from Oncomine database, in multiple cancers. The count indicates the numbers of datasets with statistically significant ($p < 0.01$) mRNA over-expression (Red) or down-expression (Blue) of CD70 or B7-H3 (cancer vs. corresponding normal tissue). Cell color is determined by the best gene rank percentile for the analysis within the cell.

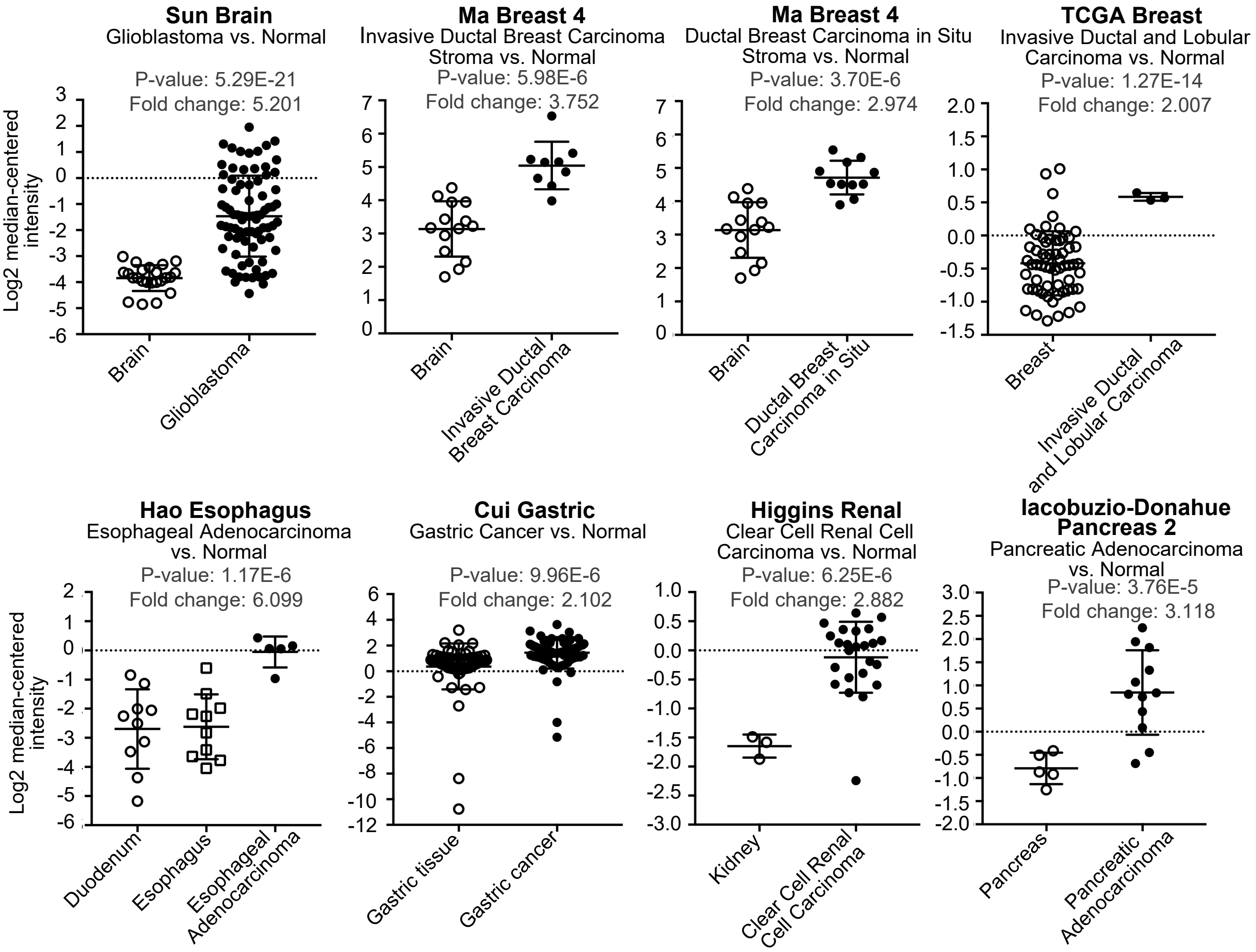

Figure S2. Transcript of B7-H3 in multiple tumors including glioblastoma, breast carcinoma, esophageal adenocarcinoma, clear cell renal cell carcinoma, and pancreatic adenocarcinoma, compared with the corresponding normal tissue, were shown based on the Oncomine database.

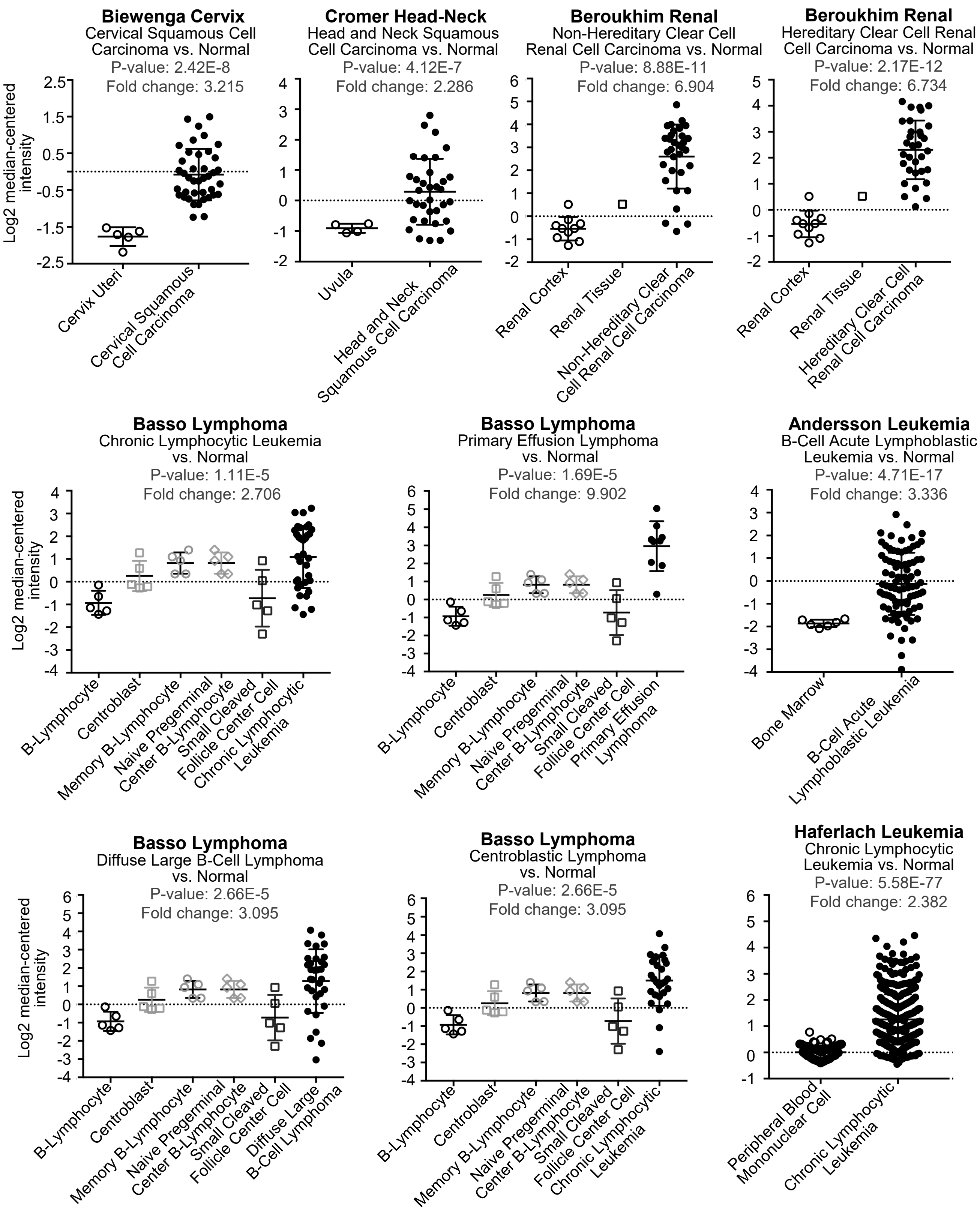

Figure S3. Transcript of CD70 in various tumors including cervical squamous cell carcinoma, head and neck squamous cell carcinoma, clear cell renal cell carcinoma, and several types of leukemia or lymphoma, compared with the corresponding normal tissue, were shown based on the Oncomine database.

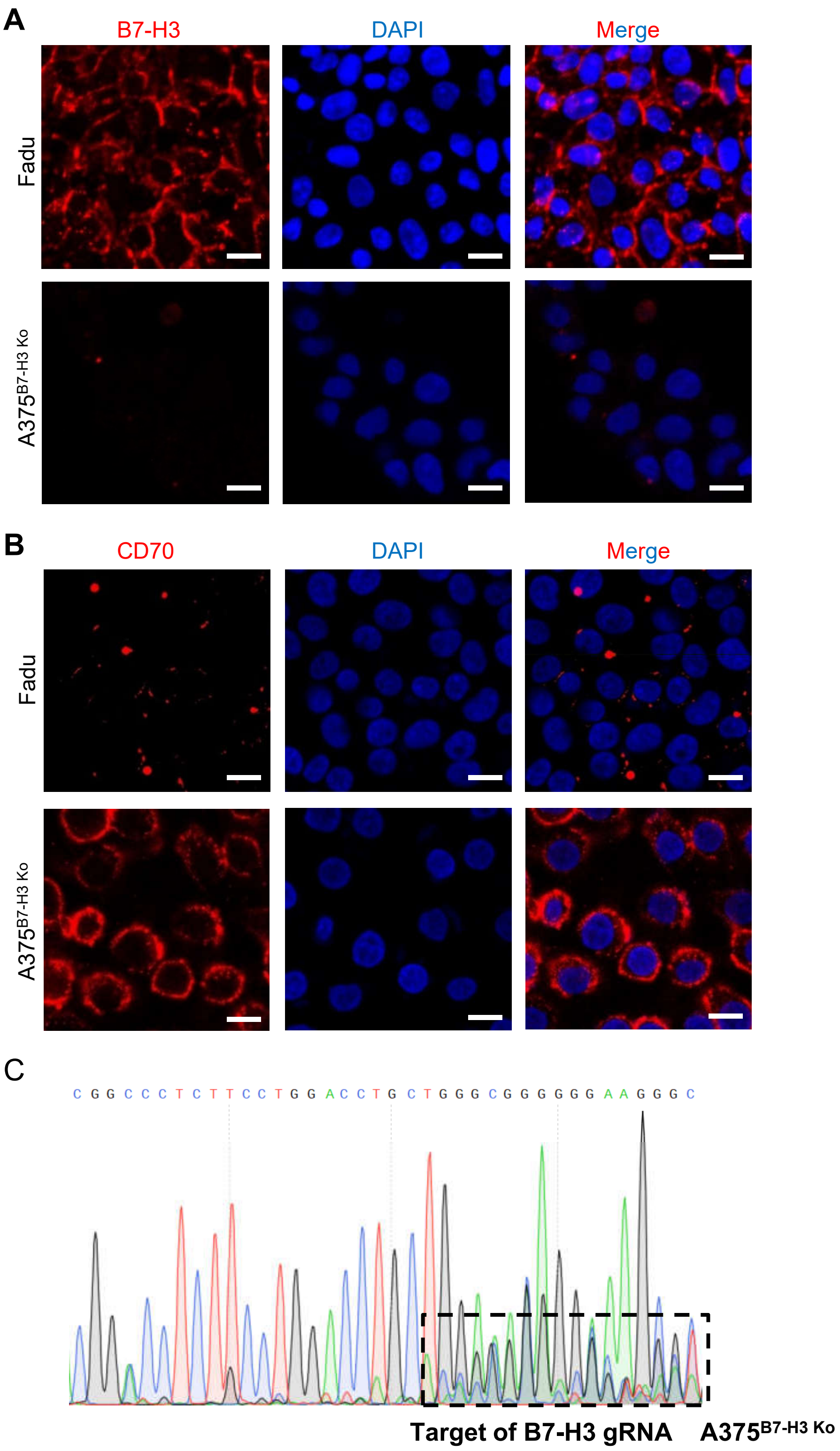

Figure S4. Expression of CD70 and B7-H3 in control tumor cell lines. (A, B) Representative images showed the immunofluorescence staining of B7-H3 and CD70 together with DAPI in Fadu and A375$^{B7\text{-}H3Ko}$ tumor cells. Scale bar: 20 μm(C) Sequencing analysis of A375$^{B7\text{-}H3Ko}$ cells indicated knocking out B7-H3 gene.

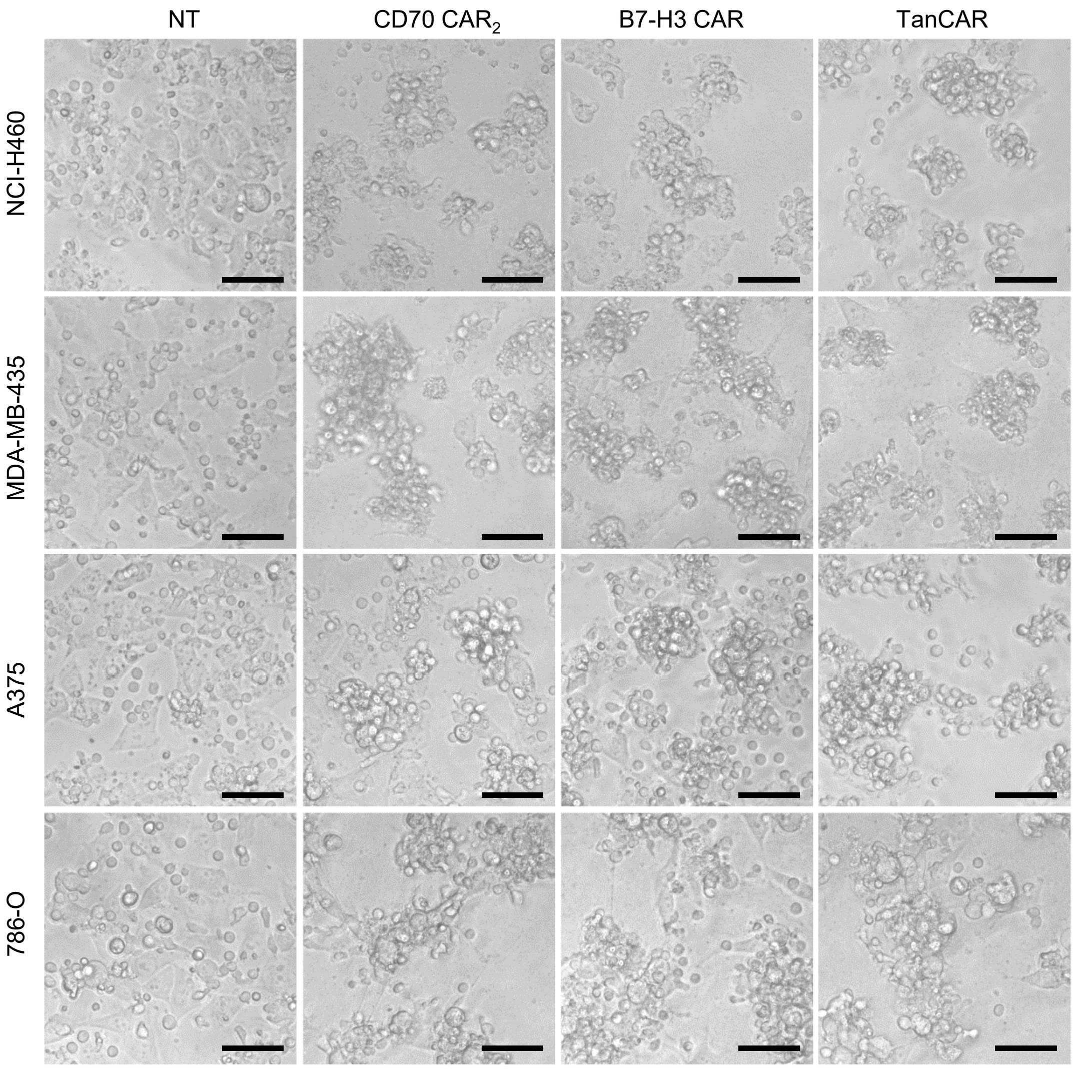

Figure S5. Images of coculture assay comparing TanCAR-T cells with control CAR-T cells when encountering NCI-H460, A375, MDA-MB-435 and 786-O cells at a ratio of 2 effector cell to 1 target cells after 24 hours co-culture. (Scale bar, 50 μm)

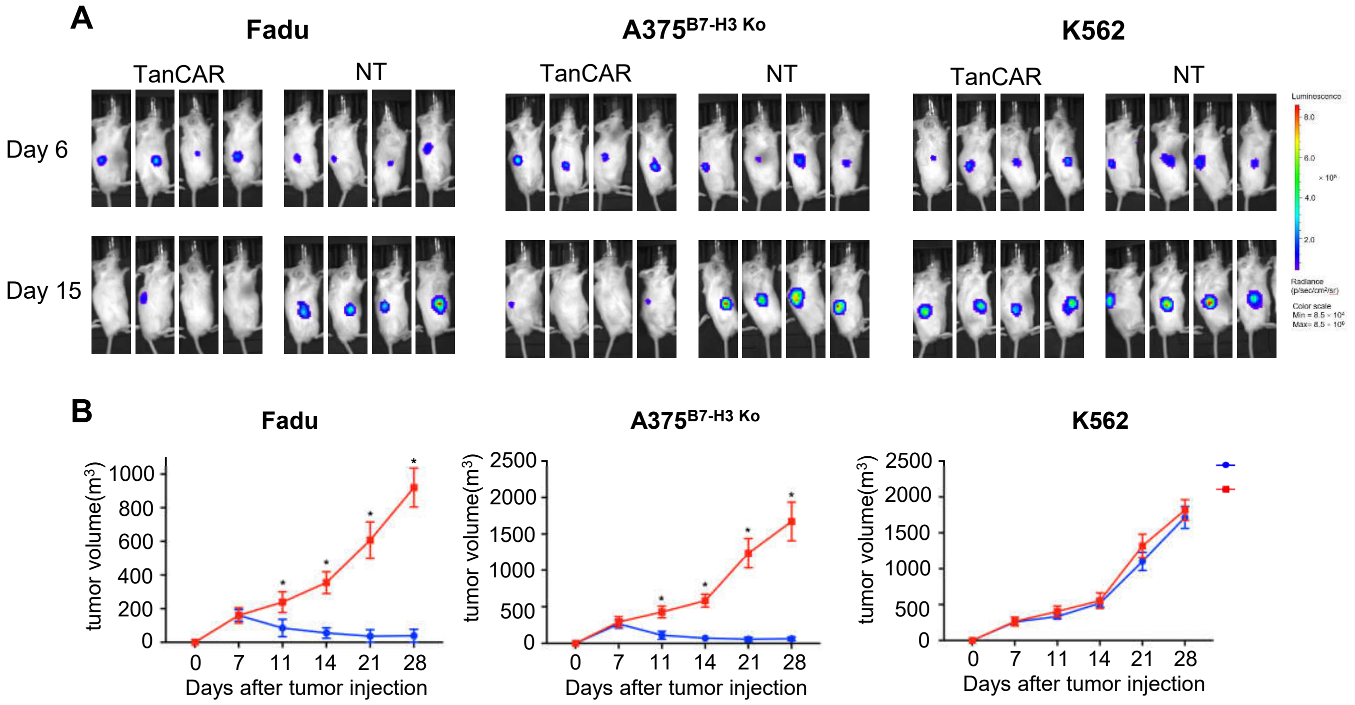

Figure S6. Antitumor response of TanCAR-T cells against $CD70^{-}/B7\text{-}H3^{+}$, $CD70^{+}/B7\text{-}H3^{-}$ and $CD70^{-}/B7\text{-}H3^{-}$ tumors in vivo. (A) Tumor bearing mice (confirmed by imaging 6 days after tumor implantation, 4/group) were adoptively transferred through tail vein injection with NT or of TanCAR-T cells (1×10^{6}/mouse) on 7 days and 10 days post tumor inoculation. Tumor growth was detected 7 days after the initial injection. (B) Tumor volume was measured at designing time points, and mean values per treated group were shown.

Table S1. IHC result of high co-expression of CD70 and B7-H3 in multiple tumor samples.

Cancer type	Total number	High co-expression of B7-H3 and CD70		High expression of B7-H3, CD70$^{-or\ low}$		High expression of CD70, B7-H3$^{-or\ low}$	
		Positive cases	rate(%)	Positive cases	rate(%)	Positive cases	rate(%)
Melanoma	5	2	40	3	60	0	0
Lung cancer	62	19	31	13	21	7	11
Kidney cancer	64	16	25	10	16	20	31
Liver cancer	15	5	33	3	20	2	13
Breast cancer	62	22	35	15	24	8	13
Esophageal cancer	32	13	40	12	37	3	9
Colon cancer	32	17	53	10	31	2	6
Glioma	34	8	24	5	15	5	15
